# Research on Remaining Useful Life Prediction Method of Rolling Bearing Based on Digital Twin

**DOI:** 10.3390/e24111578

**Published:** 2022-10-31

**Authors:** Rui Zhang, Zhiqiang Zeng, Yanfeng Li, Jiahao Liu, Zhijian Wang

**Affiliations:** School of Mechanical Engineering, North University of China, Taiyuan 030051, China

**Keywords:** digital twins, prediction of remaining useful life, neural network

## Abstract

Bearing is a key part of rotating machinery. Accurate prediction of bearing life can avoid serious failures. To address the current problem of low accuracy and poor predictability of bearing life prediction, a bearing life prediction method based on digital twins is proposed. Firstly, the vibration signals of rolling bearings are collected, and the time-domain and frequency-domain features of the actual data set are extracted to construct the feature matrix. Then unsupervised classification and feature selection are carried out by improving the self-organizing feature mapping method. Using sensitive features to construct a twin dataset framework and using the integrated learning CatBoost method to supplement the missing data sets, a complete digital twin dataset is formed. Secondly, important information is extracted through macro and micro attention mechanisms to achieve weight amplification. The life prediction of rolling bearing is realized by using fusion features. Finally, the proposed method is verified by experiments. The experimental results show that this method can predict the bearing life with a limited amount of measured data, which is superior to other prediction methods and can provide a new idea for the health prediction and management of mechanical components.

## 1. Introduction

Rolling bearing is a key component in rotating machinery, which has been widely used in modern industry [1,2]. Accurate remaining useful life estimation of bearings can significantly improve the reliability of mechanical systems, which can avoid serious failures and reduce maintenance costs. In recent years, the prediction of bearing remaining useful life (RUL) based on deep learning has made great progress [3,4,5].

Among the methods commonly used for bearing life prediction, there are often physical models and data-based methods. Physical models need to be built taking into account the influence of the complex surrounding environment and usually exhibit a weak generalization capability. Data-driven approaches, however, avoid the need for detailed modeling of complex environments and have better generalization capabilities by building models such as statistical extrapolation from historical data, and are one of the most popular research directions in the field of health prediction recently. Data-based methods often require manual extraction of features, construction of a health indicator (HI), determination of the health stage (HS), and determination of the first predicting time (FPT) before the final life expectancy can be predicted [6,7,8].

Machine learning, as a typical data-driven approach, constructs approximate models to approximate the real situation and build predictive models based on real-time, historical, and relational data. On this basis, Wang et al. proposed a residual service life prediction method for rolling bearings based on PCA and multi-dimensional feature fusion, aiming at the low reliability of bearing single feature characterization [9]. The life information of rolling bearing is characterized from many aspects, and the prediction result of residual life is more accurate and reliable. Chen et al. aimed at the problem that it is difficult to predict the bearing life under the action of a single horizontal stress [10]. A bearing life prediction method based on the failure physical reliability model is proposed to predict the bearing degradation data. Jiang et al. proposed a new dual residual attention network [11]. The hybrid extended convolutional neural network is used to learn useful features from both time and frequency directions. It provides a reliable prediction for the remaining service life of the bearing. Ahmad et al. used the adaptive prediction model based on regression to learn the evolution trend of bearing health indicators [12]. Realize accurate prediction of the remaining life of the bearing. Xu et al. proposed a hybrid model of expandable service life based on continuous monitoring and bearing condition classification [13]. The feasible parameters of bearing state quantification are evaluated, which provides an intuitive reference for the prediction of the residual life of bearings. Pan et al. proposed a two-stage prediction method for the remaining service life of bearings [14], which divided the bearing degradation into the normal stage and the degradation stage. By constructing a multivariable feedback extreme value learning machine model, the rapid prediction of the remaining useful life of the bearing is realized.

In recent years, digital twin technology has developed rapidly and has been applied in a number of practical projects [15,16,17]. Tao et al. proposed the concept of the digital twin workshop [18] and explored the application of the five-dimensional model in several fields in conjunction with practical applications. Xie et al. proposed an adaptive development environment for automotive systems based on digital twins [19], which overcomes the problems of long development cycles and poor scalability in the production process. Wei et al. proposed an optimal deployment strategy using digital twins to fully exploit the advantages of digital twins and perform tool life prediction in response to the shortcomings of current manufacturing systems [20]. Xia et al. proposed a fault diagnosis framework based on digital twins in response to the lack of fault data [21] and pre-trained the conditional data generated by digital twins to achieve accurate fault prediction. Liu et al. addressed time-varying error prediction and compensation for CNC machine tools [22] by establishing a heat transfer model for tool spindles and visualizing time-varying error models. The performance of digital twins in predicting the performance of machine tools was explored. In addition to this, digital twins have been widely used in some industrial production [23].

This paper combines digital twin with bearing life prediction and proposes a bearing residual life prediction method driven by macro and micro attention bi-directional long short-term memory (MMA-BiLSTM). Signal features are extracted from actual signals, a feature matrix is constructed, and feature selection is carried out by improving the self-organizing feature mapping method. The twin dataset framework is constructed by using sensitive features, and the missing data set is supplemented by the integrated learning CatBoost method to form a complete digital twin dataset. A new database is built to provide a qualitative analysis basis for the prediction of bearing residual life. The MMA-BiLSTM model is used for training to obtain the final residual life prediction results. The main contributions of this paper are as follows.

(1) An improved self-organizing feature mapping method is proposed, which can achieve automatic extraction of sensitive features by calculating the corresponding probability density interval of feature values;

(2) A twin data construction method is proposed to use sensitive data in the original data as a digital twin framework, and CatBoost is used to learn the remaining features and generate new digital twins;

(3) An MMA-BiLSTM neural network is proposed to extract important information through macro and micro attention mechanisms to achieve weight amplification and improve the accuracy of remaining useful life prediction.

Section 2 introduces the relevant background and theory in detail. The digital twin method and MMA-BiLASTM network proposed in this paper are given in Section 3. Finally, the effectiveness of the proposed method in this paper is demonstrated by experiments in Section 4.

## 2. Related Work

### 2.1. Self-Organizing Mapping

Self-organizing mapping (SOM) is an unsupervised learning method that can be clustered and visualized in high dimensions. On the basis of unsupervised advantage, this method can also provide the change of feature weight in the connection layer after classification. By iteratively updating the feature weight in the network structure, you can easily observe the change in the feature weight, obtain the sensitive features of the dataset classification, and provide the classification basis. SOM converts the input data into discrete low-dimensional data, which is then represented as active points in local areas or networks. After the initialization step is completed, the following three important learning processes are competition, collaboration, and adaptation.

In SOM, each neuron of the competition layer is connected according to the input N-dimensional feature vector (x) and weight (w). The range of w is between (0,1) and is initialized with any normalized value. In the learning process, calculate the distance between the feature vector x and the weight w of all neurons. When the distance is the smallest, the neuron becomes the optimal solution, which is the process of competition.

The cooperative process is that only the optimal solution of the competitive process and its neighboring neurons learn from the provided input data. In order to form a map more sensitively for similar features in the competitive hierarchy, the “optimal” neuron determines the adjacent neurons according to a fixed function, and the corresponding weight of this neuron will be updated.

The adaptive process refers to the adaptive activation function, which makes the optimal neuron and neighboring neurons more sensitive to specific input values, and also updates the corresponding weights. Through this process, the neurons adjacent to the optimal neuron will be more adaptive than those far away. The size of adaptation is controlled by the learning rate, which decreases with the learning time, and plays a role in reducing the convergence rate of SOM.

The algorithm flow of SOM is as follows:

(1) Initialization weight w; set a large initial neighborhood and set the number of network cycles;

(2) Give a eigenvector $X_{k}$: $X_{k}=\{X_{1k},X_{2k},X_{3k}\cdots X_{nk}\}$;

(3) Calculate the distance $d_{jk}$ between the feature vector $X_{k}$ and the output neuron, when $d_{jk}$ takes the minimum value, c is the optimal neuron, i.e., $x_{k}-W_{c}=\min j\{d_{jk}\}$;

(4) Update the connection weights $w_{ij}(t+1)=w_{ij}(t)+\eta (t)\{x_{i}-w_{ij}(t)\}$ of c and its domain nodes where 0<η(t)<1 is a gain function that decreases with time;

(5) Input another feature vector into the network and return to step (3) until all the feature vectors are traversed;

(6) Return to step (2) by making t=t+1, until t=T.

### 2.2. Catboost

The CatBoost algorithm is a model based on the decision tree. It does not need a large number of samples as the training data and can adapt to the training of small-scale samples and high-precision diagnosis. The CatBoost algorithm belongs to the Boosting algorithm family and is a new machine learning algorithm framework based on a gradient boosting decision tree (GBDT). The GBDT algorithm is an algorithm for regression and classification proposed by Friedman in 2000 [24], which can avoid the problem of overfitting a single decision tree due to the internal integration of multiple decision trees and the accumulation of multiple decision trees. The GBDT algorithm constructs a learner to reduce the loss along the steepest direction of the gradient at each iteration step to make up for the shortcomings of the current model.

In CatBoost, the target statistics (TS) method is usually used to process categorical features target statistic (TS) method refers to replacing category features with calculated values. Representing the ith category feature of the kth training sample as $x_{k}^{i}$, and representing the replacement target value as y. The expression of the TS method is:

(1) $${\hat{x}}_{k}^{i}=E\left(y|x^{i}=x_{k}^{i}\right)$$

The commonly used TS value calculation method is the Greedy TS method, which can be smoothed by using the average of the target variable y of the same category $x_{k}^{i}$ in training samples and using a prior probability p, expressed as:

(2) $${\hat{x}}_{k}^{i}=\frac{\sum_{j=1}^{n}II_{\left\{x_{j}^{i}=x_{k}^{i}\right\}}\cdot y_{i}+ap}{\sum_{j=1}^{n}II_{\left\{x_{j}^{i}=x_{k}^{i}\right\}}+a}$$

However, due to the duplication in the use of the training set and test set, this method will lead to condition deviation and result in overfitting. Based on this situation, CatBoost uses a method to improve the category feature processing and uses the sorting principle to solve the problem of condition shift and overfitting. In the Ordered TS, a random sequence σ is generated to number the data. The training data is selected according to the sorting principle: $D_{k}=\left\{X_{j}:\sigma (j)≺\sigma (k)\right\}$, and the test set uses all the data: $D_{k}=D$. Then the divided data set is used to calculate the Greedy TS value $x_{k}^{i}$ with a priori probability.

For multi-dimensional feature data sets, the relationship between the actual value of most features and the prediction is often nonlinear, which brings great difficulties to the analysis of feature changes. To solve this problem, the feature intersection approach is proposed, which combines different features to form new cross-features to fit the changing relationship of data set features. If all features of the dataset are crossed, the exponential dimension will grow exponentially, thus increasing the computational complexity. Based on this problem, CatBoost adopts a greedy strategy to deal with it and does not cross features at the previous node of the gradient lifting tree. Instead, the features divided in front of the node and the features within the node are considered as two groups of features to cross, and one pot vector method is used for feature fusion. The acquisition method of cross-features is expressed by the formula:

(3) $$\hat{y}=b+w_{1}{\hat{x}}_{1}+w_{2}{\hat{x}}_{2}+w_{3}{\hat{x}}_{3}$$

Where $x_{3}$ represents the sum of features of feature set $x_{1}$ and $x_{2}$, and w represents the weight relationship between feature sets. b is a constant term, which solves the problem of too many nonlinear fitting relations and cross-features. In the process of gradient promotion, different data generate different gradient classes. If the training data is repeated a lot, it will lead to gradient boosting overfitting and skew the prediction results. Based on this problem, CatBoost uses the Ordered method to sort the data sets, which reduces the error of gradient fitting. The method to generate the base evaluator is:

(4) $$h_{t}=\arg\underset{\left\{h\in H\right\}}{\min}\frac{1}{n}\overset{n}{\underset{k=1}{\sum }}\left(-g^{t}\left(x_{k},y_{k}\right)-h\left(x_{k}\right)\right)$$

Where $h_{t}$ represents the generated basis evaluator and $-g^{t}\left(x_{k},y_{k}\right)$ represents the negative gradient value of the loss function in the current gradient model. The problem of gradient lifting prediction migration is solved by the Ordered method.

CatBoost uses a symmetric tree structure in the decision tree structure. The advantage of the symmetric tree structure is that it is not easy to overfit, and it is much faster than the gradient lifting algorithm, such as XGBoost. In addition, the CatBoost algorithm can realize multiple graphics processing unit (GPU) operations. The distributed learning tree enables CatBoost to perform parallel computing, thus improving its overall computing speed.

### 2.3. BiLSTM

**Figure 1:**
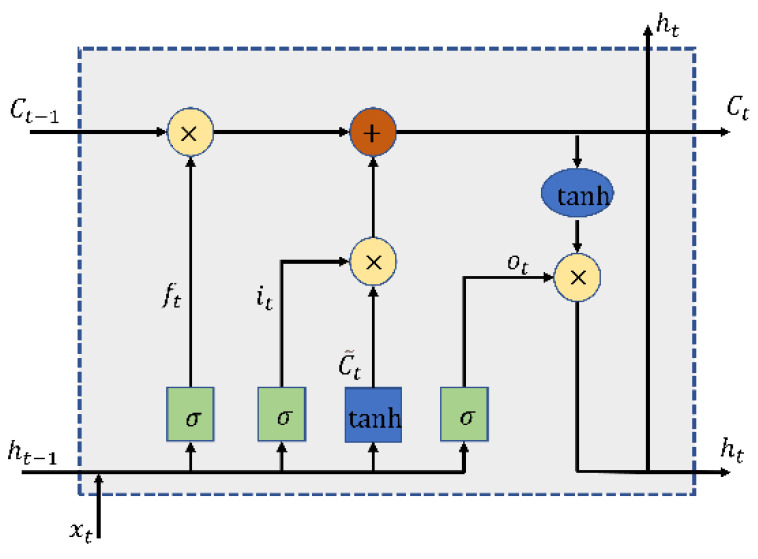
Structure of LSTM.

LSTM network is an improvement in recurrent neural network (RNN). The key to the LSTM network is the cell state. The information in the cell state is updated and deleted through the forgetting gate, update gate, and output gate. The structure of LSTM is shown in Figure 1. The following is the representation of the three gates of LSTM:

(5) $$f_{t}=\sigma \left(W_{f}\times \left[h_{t-1},x_{t}\right]+b_{f}\right)$$

(6) $$i_{t}=\sigma \left(W_{i}\times \left[h_{t-1},x_{t}\right]+b_{i}\right)$$

(7) $${\tilde{C}}_{t}=\tanh\left(W_{C}\times \left[h_{t-1},x_{t}\right]+b_{C}\right)$$

(8) $$C_{t}=f_{t}\times C_{t-1}+i_{t}\times \tilde{C}$$

(9) $$o_{t}=\sigma \left(W_{o}\times \left[h_{t-1},x_{t}\right]+b_{o}\right)$$

(10) $$h_{t}=o_{t}\times \tanh\left(C_{t}\right)$$

Because the next moment prediction output of unidirectional LSTM is only affected by the previous multiple time inputs, in many cases, the prediction will be affected by the previous and subsequent multiple time inputs at the same time. In order to fully extract the correlation between before and after features and obtain better prediction results. The BiLSTM network was introduced to calculate the front and back information from two opposite directions (forward network output is ${\vec{h}}_{t}$, backward network output is $\overset{\leftarrow }{h_{t}}$):

(11) $${\vec{h}}_{t}=\mathrm{LSTM}(x_{t},\vec{h_{t-1}})$$

(12) $$\overset{\leftarrow }{h_{t}}=\mathrm{LSTM}(x_{t},\overset{\leftarrow }{h_{t+1}})$$

Finally, output the comprehensive result of the result stack of the forward network layer and the backward network layer.

## 3. Prediction of Bearing RUL Based on Digital Twin

Aiming at the complex working environment of special mechanical equipment, data collection is difficult, and the amount of data is small. This paper presents a method for predicting the remaining service life of small sample bearings based on data twin driving. First of all, feature extraction is carried out on the actual data to form a high-dimensional feature dataset. Then, an improved self-organizing feature mapping method (ISOFM) is used to select features, calculate the numerical probability density intervals of features corresponding to sensitive features, determine the optimal number of sensitive features, and form a feature framework. The feature framework is combined with existing data to form an interactive dataset with missing data, and CatBoost integrated learning algorithm is introduced. The missing eigenvalues are taken as the feature learning objectives of CatBoost, respectively, and their regression operation characteristics are used to complement the interactive dataset, thus forming a complete twin dataset. Finally, the macro and micro attention mechanisms are combined with BiLSTM to form MMA-BiLSTM. The weight of MMA-BiLSTM is amplified in the whole time dimension and each time dimension to realize the residual life prediction of bearings.

### 3.1. ISOFM

The classical SOM method needs to preset the number of nodes in the output layer. Therefore, it is necessary to improve this method to make it have the ability to adaptively select the number of nodes in the output layer. In this paper, a generation-by-generation node processing method is proposed to remove nonsensitive features in the neighborhood during SOM’s selection of sensitive features. Both the number of output nodes of SOM can be adaptively obtained, and the nonsensitive feature removal strategy can also improve the feature selection efficiency of SOM. The proposed ISOFM method mainly selects nodes by introducing a learning rate α and a relative removal rate parameter β. Set the Euclid distance and the threshold value of the feature node weight to improve. The improved weight update formula is:

(13) $$\overset{\leftarrow }{h_{t}}=\mathrm{LSTM}(x_{t},\overset{\leftarrow }{h_{t+1}})$$

(14) $$W_{j}(t+1)=\lambda \left[W_{j}(t)+\alpha (t)\left(X_{i}-W_{j}(t)\right)\right]$$

(15) $$\lambda =\{\begin{array}{l} 0,\frac{(1-\beta ){\left[\max\left(d_{j}\right)-\min\left(d_{j}\right)\right]}^{2}}{4{\left(d_{j}-\sum_{j=1}^{m}d_{j}/J\right)}^{2}}<1;\\ 1,\frac{(1-\beta ){\left[\max\left(d_{j}\right)-\min\left(d_{j}\right)\right]}^{2}}{4{\left(d_{j}-\sum_{j=1}^{m}d_{j}/J\right)}^{2}}\geq 1 \end{array}$$

### 3.2. MMA-BiLSTM

The derivation of BiLSTM based on macro and micro attention mechanisms is as follows: macro and micro attention mechanisms refer to the operation of the attention mechanism on the whole time dimension of input data and data on each time dimension. Specifically, firstly, the data matrix generated by digital twins is processed, and its macro and micro attention coefficients are calculated using MMA. In the prediction process, the input dataset of the whole time dimension is $X_{t}=\left[\begin{array}{cccc} x_{1}, & x_{2}, & \ldots & ,x_{t} \end{array}\right]$. Where $x_{t^{\prime }}={\left[\begin{array}{cccc} x_{t^{\prime },1} & x_{t^{\prime },2} & \ldots & x_{t^{\prime },n} \end{array}\right]}^{T}$ represents the input data at time $t^{\prime }$, and the macro attention mechanism processes the data in the whole time dimension through the attention mechanism; the micro attention mechanism is to use the attention mechanism to process input data $x_{t^{\prime }}$ in each time dimension [25].

The formula for calculating macro and micro attention coefficients:

(16) $$\chi_{t^{\prime }}=\frac{\exp\left(S\left({\bar{x}}_{t^{\prime }},q_{M}\right)\right)}{\sum_{j=1}^{t}\exp\left(S\left({\bar{x}}_{j},q_{M}\right)\right)}$$

(17) $$\alpha_{t^{\prime },i}=\frac{\exp\left(s\left(x_{t^{\prime },i},q_{t^{\prime },m}\right)\right)}{\sum_{j=1}^{n}\exp\left(s\left(x_{t^{\prime },j},q_{t^{\prime },m}\right)\right)+\sum_{p=1}^{m}\exp\left(s\left(h_{t^{\prime }-1,p},q_{t^{\prime },m}\right)\right)}$$

Where $\alpha_{t^{\prime },i}$ is the attention coefficient of input data in the micro attention mechanism. $\chi_{t^{\prime }}$ is the macro attention coefficient obtained in the whole time dimension. ${\bar{x}}_{t^{\prime }}$ is the mean value of $x_{t^{\prime }}$, $x_{t^{\prime },j}$ is the j element in the input data $x_{t^{\prime }}={\left[\begin{array}{cccc} x_{t^{\prime },1} & x_{t^{\prime },2} & \ldots & x_{t^{\prime },n} \end{array}\right]}^{T}$ at the time of t, t is the dimension of the input dataset $X_{t}=\left[\begin{array}{cccc} x_{1}, & x_{2}, & \ldots & ,x_{t} \end{array}\right]$, ${\bar{x}}_{j}$ is the mean value of the j vector in the input dataset $X_{t}$, and q is the query vector. In the MMA-BiLSTM network training process, set the macro level query vector $q_{M}$ and the micro level query vector $q_{m}$; the relevant scoring function is calculated as follows:

(18) $$S\left({\bar{x}}_{j},q_{M}\right)=\frac{{\bar{x}}_{j}q_{M}}{\sqrt{t}}$$

(19) $$s\left(x_{t^{\prime },j},q_{t^{\prime },m}\right)=\frac{x_{t^{\prime },j}^{T}q_{t^{\prime },m}}{\sqrt{n+m}}$$

Where n is the dimension of input data $x_{t^{\prime }}={\left[\begin{array}{cccc} x_{t^{\prime },1} & x_{t^{\prime },2} & \ldots & x_{t^{\prime },n} \end{array}\right]}^{T}$ at time t.

According to the corresponding macro and micro attention coefficients, the associated input data weights and recursive data weights are magnified at multiple levels.

(20) $$w_{t^{\prime },ix}^{\prime }=\left(1+\chi_{t^{\prime }}\right)\times w_{t^{\prime },ix}(\alpha_{t^{\prime }}+1)$$

(21) $$w_{t^{\prime },ox}^{\prime }=\left(1+\chi_{t^{\prime }}\right)\times w_{t^{\prime },ox}(\alpha_{t^{\prime }}+1)$$

(22) $$w_{t^{\prime },fx}^{\prime }=\left(1+\chi_{t^{\prime }}\right)\times w_{t^{\prime },fx}(\alpha_{t^{\prime }}+1)$$

Wherein, $w_{t^{\prime },ix}$ represents the weight between the input data of the BiLSTM neural network and the input gate in the hidden layer, $w_{t^{\prime },ox}$ represents the weight between the input data of the BiLSTM neural network and the output gate in the hidden layer, $w_{t^{\prime },fx}$ represents the weight between the input data of the LSTM neural network and the forgetting gate in the hidden layer, $w_{t^{\prime },ix}^{\prime }$ represents the weight between the input data of the MMA-BiLSTM neural network and the input gate in the hidden layer, $w_{t^{\prime },ox}^{\prime }$ represents the weight between the input data of MMA-BiLSTM neural network and the output gate in the hidden layer, and $w_{t^{\prime },fx}^{\prime }$ represents the weight between the input data of MMA-BiLSTM neural network and the forgetting gate in the hidden layer.

According to the amplification of input data weight and recursive number weight, the corresponding calculation results are obtained:

(23) $$f_{t^{\prime }}=\sigma \left(w_{t^{\prime },f}^{\prime }\left[x_{t^{\prime }},h_{t^{\prime }-1}\right]+b_{f}\right)$$

(24) $$i_{t^{\prime }}=\sigma \left(w_{t^{\prime },i}^{\prime }\left[x_{t^{\prime }},h_{t^{\prime }-1}\right]+b_{i}\right)$$

(25) $${\tilde{c}}_{t^{\prime }}=\tanh\left(w_{t^{\prime },c}\left[x_{t^{\prime }},h_{t^{\prime }-1}\right]+b_{c}\right)$$

(26) $$c_{t^{\prime }}=f_{t^{\prime }}\times c_{t^{\prime }-1}+i_{t^{\prime }}\times {\tilde{c}}_{t^{\prime }}$$

(27) $$o_{t^{\prime }}=\sigma \left(w_{t^{\prime },o}^{\prime }\left[x_{t^{\prime }},h_{t^{\prime }-1}\right]+b_{o}\right)$$

(28) $$h_{t^{\prime }}=o_{t^{\prime }}\times \tanh(c_{t^{\prime }})$$

(29) $${\vec{h}}_{t^{\prime }}=\mathrm{LSTM}(x_{t^{\prime }},\vec{h_{t^{\prime }-1}})$$

(30) $$\overset{\leftarrow }{h_{t^{\prime }}}=\mathrm{LSTM}(x_{t^{\prime }},\overset{\leftarrow }{h_{t^{\prime }+1}})$$

(31) $$F_{t^{\prime }}=g\left(W_{t^{\prime },{\vec{h}}_{y}}{\vec{h}}_{t^{\prime }}+W_{{\overset{\leftarrow }{t^{\prime },h}}_{y}}{\overset{\leftarrow }{h}}_{t^{\prime }}+b_{y}\right)$$

Wherein, σ is sigmoid activation function, g is linear activation function, $b_{i}$ is MMA-BilLSTM hidden layer input gate offset term, $b_{f}$ is MMA-BiLSTM hidden layer forgetting gate offset term, $b_{c}$ is MMA-BiLSTM hidden layer storage cell unit offset term, $b_{o}$ is MMA-BiLSTM hidden layer output gate offset term, $b_{y}$ is MMA-BiLSTM output layer offset term, $i_{t^{\prime }}$ is the input gate output at $t^{\prime }$ time, $f_{t^{\prime }}$ is the forgetting gate output at $t^{\prime }$ time, $c_{t^{\prime }}$ is the storage cell unit output at $t^{\prime }$ time, and $F_{t^{\prime }}$ is the output layer output at $t^{\prime }$ time.

**Figure 2:**
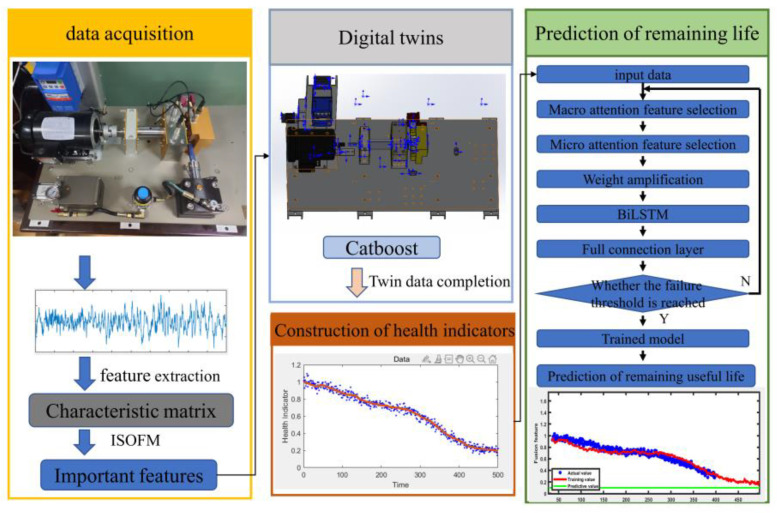
Flowchart of the proposed method.

The bearing vibration data are collected separately as samples, and the data samples are twin expanded. Finally, different machine learning methods are used to compare the prediction accuracy between the original sample and the interactive dataset. The specific process and structure of the proposed method can be shown in Figure 2. The specific steps of the proposed method are shown as follows:

(1) Set up a test platform to collect vibration signals of bearings from normal operation to fault status;

(2) Extraction of time-domain and frequency-domain features of vibration signals from the original signal;

(3) Use ISOFM to determine the number of sensitive features and select features from the acquired feature data set, and extract the main features in the feature set that can determine the signal category;

(4) The probability density distribution models of sensitive features in feature data sets are constructed, respectively; determine the feature frame and the selection range of its feature values;

(5) The feature data frame generated is combined with the feature data set extracted from the initial samples interactively, and the data at the nonsensitive features are represented by missing values;

(6) The CatBoost regression algorithm is used to fill in the missing values in the interactive dataset containing missing values. Sorted according to importance, the missing value is used as the prediction target to fill the characteristic value. During the filling process, the missing values of other features are filled with the feature mean value;

(7) An interactive dataset with a complete data structure is obtained, i.e., a twin feature dataset that expresses vibration signal fault information obtained from a small amount of data. The dataset is normalized to fit the health indicators of the bearing;

(8) Use the first k health indicators of the bearing as network input to predict the health value at moment k + 1;

(9) Repeat step 8 a certain number of times, and when these output values are less than 0, the inverse normalization of the sampled points results in RUL.

## 4. Experimental Validation

### 4.1. Experimental Description

**Figure 3:**
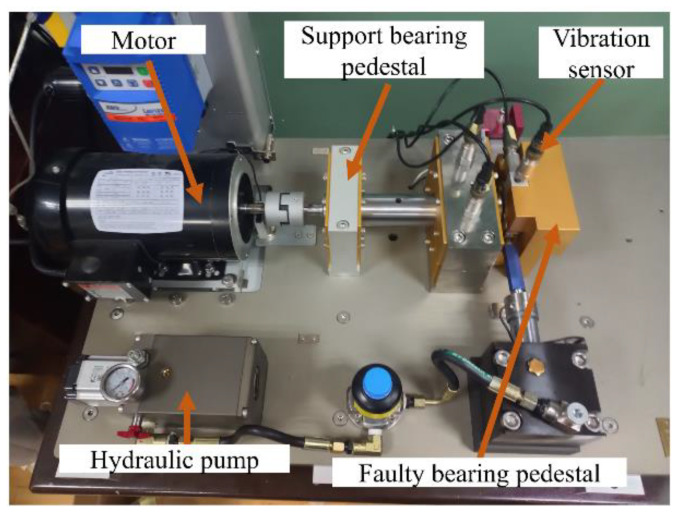
Bearing life test bench.

**Figure 4:**
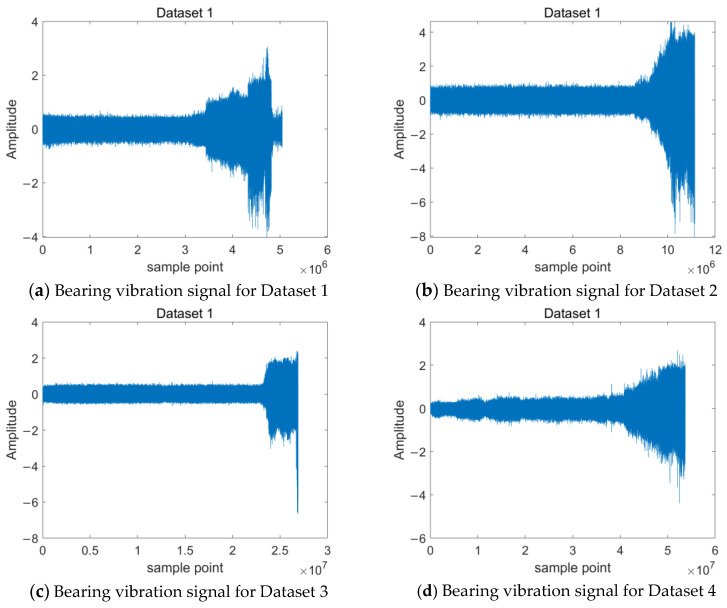
Bearing vibration signal.

The experimental design was carried out according to the research idea shown in Figure 2, and tests were conducted on a full life-bearing fatigue test machine. The main structure of the platform is the motor, supporting bearing housing, vibration sensor, hydraulic resistor, coupling, and other mechanisms. The experimental setup is shown in Figure 3. The tests were carried out at different rotational speed conditions, and the test was set up with a sampling frequency of 25.6 kHz, a sampling interval of 1 min, and a duration of 1.28 s per sample. The bearing vibration signal is shown in Figure 4. The experimental data are described in Table 1.

**Figure 5:**
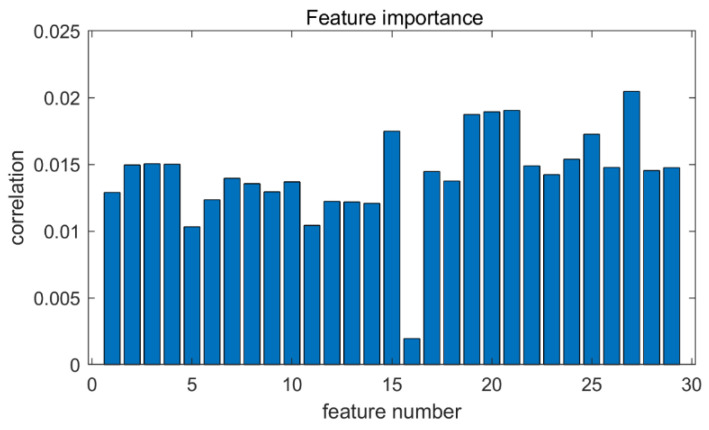
Feature importance diagram.

Corresponding to the data sets in Table 1, the signal features for the different operating conditions were obtained by pre-processing. These include 13 time-domain features and 16 frequency-domain features, which are combined into a feature matrix. The details are as follows: (1) maximum value, (2) minimum value, (3) median, (4) mean, (5) peak difference, (6) mean of absolute values, (7) variance, (8) standard deviation, (9) cliffness, (10) skewness, (11) root mean square, (12) impulse factor, (13) margin factor, (14) amplitude maximum, (15) amplitude minimum, (16) amplitude median, (17) amplitude mean, (18) amplitude peak difference, (19) amplitude peak threshold, threshold of 75% of the amplitude peak difference, (20) amplitude peak, (21) amplitude peak corresponding frequency, (22) frequency center of gravity, (23) mean square frequency (24) frequency variance, (25) frequency standard deviation, (26) short time power spectral density, (27) spectral entropy, (28) fundamental frequency, and (29) resonance peak. The initial feature dataset was processed using ISOFM. Five groups of sensitive features were finally retained adaptively by the algorithm, and the feature importance is shown in Figure 5. In the experiment, the learning rate and removal rate are set to a = 0.1 and b = 0.1, respectively, and the number of iterations of the algorithm is set to 100.

In constructing the digital twin data feature framework, the optimal number of sensitive features is first determined, and the feature framework is formed. The feature framework is combined with existing data to form an interactive dataset with missing data. The CatBoost integrated learning algorithm is introduced, and the missing feature values are used as the feature learning targets for CatBoost, respectively. The interaction dataset is complemented by using its regression operation properties to form a complete twin dataset. In this paper, the rolling bearing vibration signals are used as samples, the data samples are twinned and expanded, and finally, the prediction errors of the original samples are compared with the interaction dataset.

### 4.2. Comparison of Digital Twin Data with Initial Data

**Figure 6:**
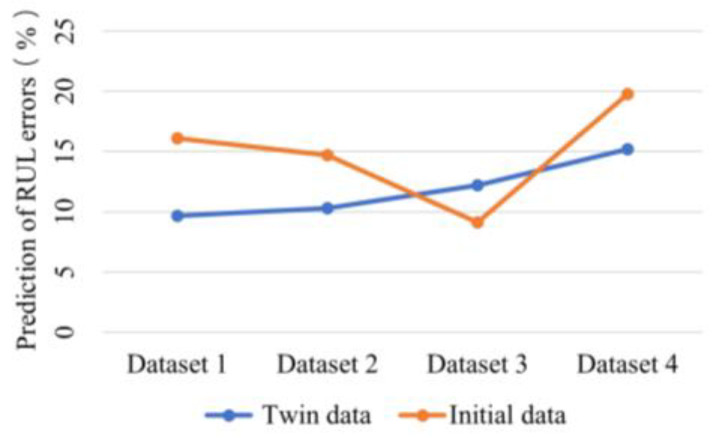
Comparison of prediction errors between initial data and twin data.

After generating the digital twin interaction dataset, the initial data and the digital twin-generated data were used separately for bearing life prediction. The dataset was validated for prediction by an LSTM network, and the first 80% of the normalized sample points were used as the training set for predicting the RUL. The current sample points and true RULs for Dataset 1, Dataset 2, Dataset 3, and Dataset 4 are shown in Table 2. The average error results obtained from multiple cross-validations of the different data are placed in Figure 6 for comparison.

It can be seen from Figure 6 that the prediction errors for the Dataset 1, Dataset 2, and Dataset 4 twin data are smaller than the original data. In Dataset 3, the original data prediction error is smaller, and the two are closer. The experimental results validate the effectiveness of the twin dataset. The twin dataset produced by constructing new feature data and fusing it with the initial dataset clearly has better predictive power than the initial dataset.

### 4.3. MMA-BiLSTM

**Figure 7:**
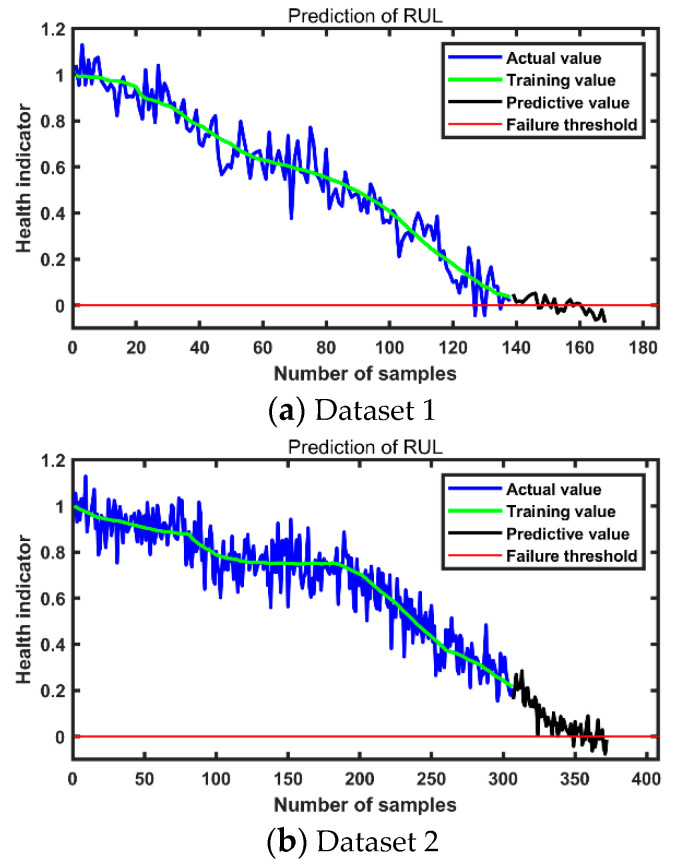

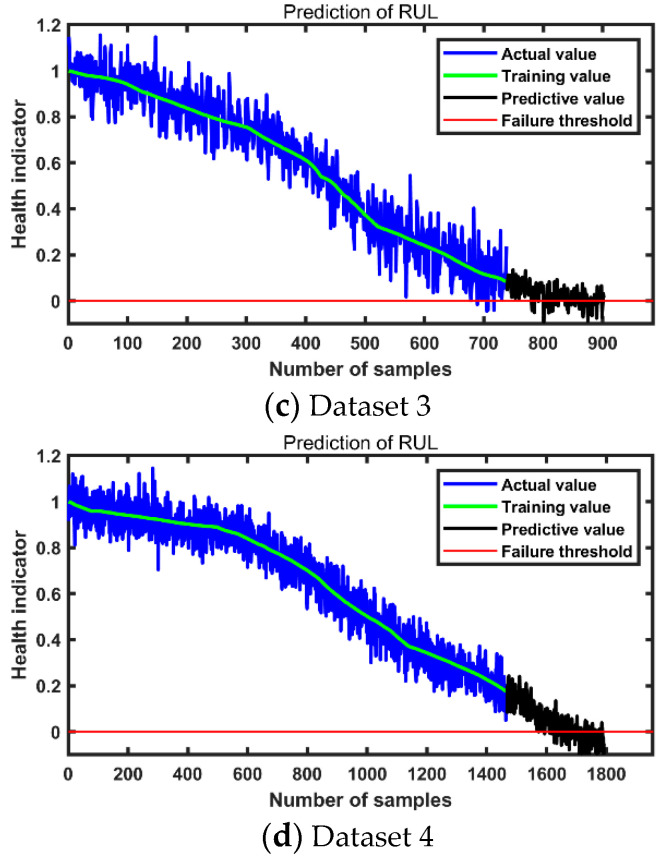
RUL of bearings predicted by MMA-BiLSTN.

To improve the prediction performance of LSTM networks. The macro-microscopic attention mechanism is combined with BiLSTM to propose MMA-BiLSTM. The method is compared with BiLSTM, LSTM, and GRU for experiments. The results are presented in Table 3, Table 4, Table 5 and Table 6. where dataset a-b represents the ath dataset and the bth experiment. The experimental data are in seconds. The number of input units and output units in MMA-BiLSTM are set to 32 and 1, respectively, and the learning rate is set to 0.01. In this research, the number of hidden layer units is set to 128. the initialization method of the neural network uses standard initialization. The MMA-BiLSTN predictions for different data sets are shown in Figure 7. The mean absolute error (MAE) and root mean square error (RMSE) were used to evaluate the prediction effect. They are defined as follows.

(32) $$\mathrm{MAE}=\frac{1}{m}\overset{m}{\underset{i=1}{\sum }}{\left(rul_{i}-ru{\hat{l}}_{i}\right)}^{2}$$

(33) $$\mathrm{RMSE}=\sqrt{\frac{1}{m}\overset{m}{\underset{i=1}{\sum }}{\left(rul_{i}-ru{\hat{l}}_{i}\right)}^{2}}$$

**Figure 8:**
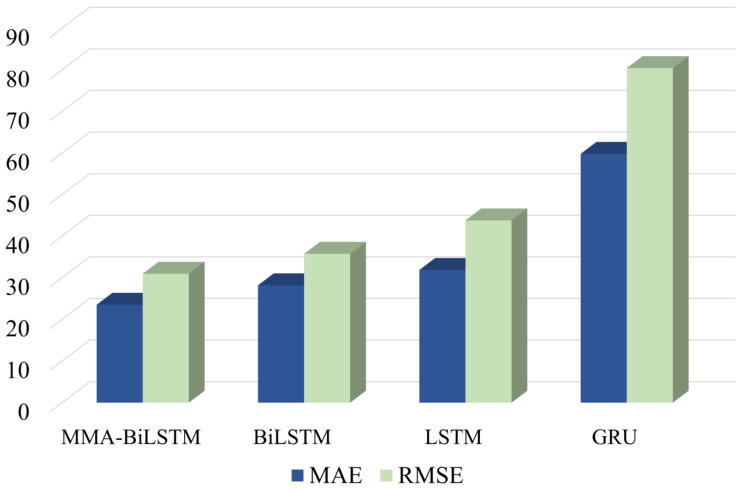
Comparison of MAE and RMSE predicted by different models.

From Table 3, Table 4, Table 5 and Table 6, it can be concluded that compared with BiLSTM, LSTM, GRU, and MMA-BiLSTM has a smaller prediction error. This shows the advantages of data generated by digital twins. In Figure 8, MAE and RMSE predicted by the MMA-BiLSTM method are smaller than those predicted by other methods. It shows the suitable performance of MMA-BiLSTM in RUL prediction.

The prediction performance of the methods proposed in this paper has been improved, and the predicted values of RUL for bearings in different working conditions are closer to the actual values than other methods, which can provide an effective way to predict bearing life.

## 5. Conclusions

For the bearing life prediction problem, this paper proposes a bearing life prediction method combining digital twin and MMA-BiLSTM network. Firstly, an extracted sensitive feature matrix is constructed to build the digital twin framework; the data set is supplemented by the integrated learning CatBoost method for missing data to form a complete digital twin data set. The MMA-BiLSTM network is proposed for life prediction. Finally, the accuracy of the proposed approach was verified by building a bearing life prediction test bench. The method can be further extended and applied to other condition parameters of gearboxes to provide data closer to the true value for predicting the RUL of machinery.

## Figures and Tables

**Table 1:** Description of experimental data.

	Dataset 1	Dataset 2	Dataset 3	Dataset 4
Load (kg)	500	1000	1000	1000
Speed (rpm)	1200	2100	2100	2100
Time (min)	154	340	821	1639

**Table 2:** Comparison of digital twin data with initial data.

Data Set	Current Sample Point	Real RUL	Prediction of RUL Errors (%)	
			Initial Data	Twin Data
Dataset 1	123	31	16.12	9.67
Dataset 2	272	68	14.7	10.29
Dataset 3	657	164	9.14	12.19
Dataset 4	1311	328	19.8	15.2

**Table 3:** RUL prediction results and comparison for Dataset 1.

	MMA-BiLSTM	BiLSTM	LSTM	GRU
Dataset 1-1	145	164	158	231
Dataset 1-2	143	141	137	164
Dataset 1-3	152	139	156	169
Dataset 1-4	146	153	122	152
MAE	7.50	9.75	13.75	26.0
RMSE	8.21	11.12	18.25	39.55

**Table 4:** RUL prediction results and comparison for Dataset 2.

	MMA-BiLSTM	BiLSTM	LSTM	GRU
Dataset 2-1	327	357	364	390
Dataset 2-2	365	384	332	367
Dataset 2-3	350	363	359	471
Dataset 2-4	333	308	326	361
MAE	13.75	29.0	16.25	57.25
RMSE	15.35	30.73	17.29	72.16

**Table 5:** RUL prediction results and comparison for Dataset 3.

	MMA-BiLSTM	BiLSTM	LSTM	GRU
Dataset 3-1	820	827	780	941
Dataset 3-2	768	835	773	893
Dataset 3-3	795	758	826	754
Dataset 3-4	832	780	841	807
MAE	22.75	31.0	28.50	68.25
RMSE	30.03	38.75	33.21	77.89

**Table 6:** RUL prediction results and comparison for Dataset 4.

	MMA-BiLSTM	BiLSTM	LSTM	GRU
Dataset 4-1	1712	1679	1726	1738
Dataset 4-2	1691	1648	1694	1704
Dataset 4-3	1652	1712	1689	1670
Dataset 4-4	1678	1707	1746	1695
MAE	44.25	47.5	74.75	62.75
RMSE	49.30	53.93	78.33	67.31

## Data Availability

Not applicable.
